# Foliar Application of Calcium and Growth Regulators Modulate Sweet Cherry (*Prunus avium* L.) Tree Performance

**DOI:** 10.3390/plants9040410

**Published:** 2020-03-26

**Authors:** Sofia Correia, Filipa Queirós, Helena Ferreira, Maria Cristina Morais, Sílvia Afonso, Ana Paula Silva, Berta Gonçalves

**Affiliations:** 1 Centre for the Research and Technology of Agro-Environmental and Biological Sciences (CITAB), University of Trás-os-Montes e Alto Douro, 5000-801 Vila Real, Portugal; helenaf@utad.pt (H.F.); cmorais@utad.pt (M.C.M.); safonso@utad.pt (S.A.); asilva@utad.pt (A.P.S.); bertag@utad.pt (B.G.); 2 National Institute for Agrarian and Veterinary Research (INIAV, I.P.), Pólo de Alcobaça, Estrada de Leiria, 2460-059 Alcobaça, Portugal; filipa.queiros@iniav.pt

**Keywords:** spray treatments, leaf gas exchange, leaf metabolites, water status, yield, cherry cracking

## Abstract

Cracking of sweet cherry (*Prunus avium* L.) fruits is caused by rain events close to harvest. This problem has occurred in most cherry growing regions with significant economic losses. Several orchard management practices have been applied to reduce the severity of this disorder, like the foliar application of minerals or growth regulators. In the present study, we hypothesized that preharvest spray treatments improve the physiological performance of sweet cherry trees and could also mitigate environmental stressful conditions. Effects of repeated foliar spraying of calcium (Ca), gibberellic acid (GA_3_), abscisic acid (ABA), salicylic acid (SA), glycine betaine (GB), and the biostimulant *Ascophyllum nodosum* (AN) on the physiological and biochemical performance of ‘Skeena’ sweet cherry trees during two consecutive years (without Ca in 2015 and in 2016 with addition of Ca) were studied. Results showed that in general spray treatments improved the physiological performance and water status of the trees. AN and ABA sprays were demonstrated to be the best compounds for increasing yield and reducing cherry cracking as well as improving photosynthetic performance and leaf metabolites content. In conclusion, AN and ABA might be promising tools in the fruit production system.

## 1. Introduction

The most recent climate projections [1] point to a decrease in water availability, an increase in air temperature, and the occurrence of extreme phenomena, such as excessive rainfall near the harvest period, which may increase the incidence of fruit cracking in sweet cherry (*Prunus avium* L.). Consequently, significant economic losses occur, due to a strong reduction of the commercial value of the cherries. Under the current climate changing scenario [1] and also due to the increase of global trade in fruit to meet consumer demand for regular supply of high quality fruit, it is important to understand the relationship between preharvest treatments with calcium (Ca) and growth regulators and the physiological parameters of sweet cherry trees. This information can provide new insights into the putative potential measures to mitigate environmental stressful conditions.

Ca is an important macronutrient, which is involved in the regulation of the main physiological processes in plants, contributing to the strength of the cell walls and membranes and reducing cherry cracking [2,3,4,5,6]. Under drought conditions, growth and physiological performance are improved by Ca sprays of *Zoysia japonica* and *Zea mays* plants [7,8]. Gibberellic acid (GA_3_) has been used as a compound to promote growth, which regulates plant growth processes, like seed germination, flower, and fruit development [9,10,11]. However, the impact of GA_3_ spray treatment on fruit cracking incidence is sometimes contradictory [12,13,14,15,16]. Other plant hormones, like salicylic acid (SA) and abscisic acid (ABA), are signaling phytohormones with different regulatory roles in plant metabolism and adaptation to abiotic stresses [17,18]. The yield increase in olive [19], peach [20], and strawberry [21] is associated with SA application, as well as the quality improvement of cherry fruits [22,23]. ABA stimulates stomatal closure and minimizes water loss by transpiration [24]. Additonally, Balbontín et al. [25] mentioned that ABA foliar sprays reduced cracking in ‘Bing’ cherries. Therefore, exogenous ABA application can have a great interest in water conservation in agricultural settings. 

Although no consistent literature is available about the effect of preharvest substances, such as glycine betaine (GB) and *Ascophyllum nodosum* (AN), on the physiological performance of sweet cherry trees, these compounds might be a new and innovative solution to increase the crop ability to tolerate stressful environments. The accumulation of osmolytes such as GB (quaternary ammonium compound) in cells can stabilize the structures by maintaining the integrity of membranes against the damaging effects of abiotic stresses via osmoregulation or osmoprotection [26]. Seaweed based biostimulants, like AN, are composed of several components, such as plant hormones, proteins, sugars, vitamins, humic substances, and phenolic compounds [27,28]. Several published reports suggest that biostimulants improve plant productivity by increasing the minerals assimilation and the photosynthetic rate, reducing the transpiration and the fruit cracking incidence [27,29,30,31]. Despite these well-documented effects, no consistent results are yet available, at least to our knowledge, about the influence of plant growth regulators, with the addition or no addition of Ca, on the performance of sweet cherry trees.

Therefore, the objective of this study was to assess the effect of plant growth regulators, with the addition or no addition of Ca, on the plant physiological and biochemical responses, namely plant water status, photosynthetic performance, and leaf metabolites, as well as on the yield of sweet cherry trees and cherry cracking incidence.

## 2. Material and Methods

### 2.1. Experimental Site and Plant Material 

Experiments were conducted in Carrazedo de Montenegro, Portugal (latitude 41°33′ N, longitude 7°17′ W, altitude 682 m), in 2015 and 2016, on a six-year-old late-maturing ‘Skeena’ sweet cherry orchard grafted on ‘Gisela 6’ rootstock. The soil characteristics were: 13 g kg^−1^ of organic matter content, high K_2_O (125 mg kg^−1^) and medium P_2_O_5_ (75 mg kg^−1^) contents, medium texture, and pH 5.5. Trees were trained under a vertical axis system with a spacing of 4.5 m between rows and 2.0 m in the row (about 864 trees ha^−1^) [32]. Between May and September, trees were daily drip-irrigated for 4 h per day (drippers 1 m apart in line with a 4 L h^−1^ flow rate) and summer pruned. According to recommendations provided by a certified soil analysis laboratory of University of Trás-os-Montes e Alto Douro (UTAD), trees were also periodically fertilized. 

**Figure 1:**
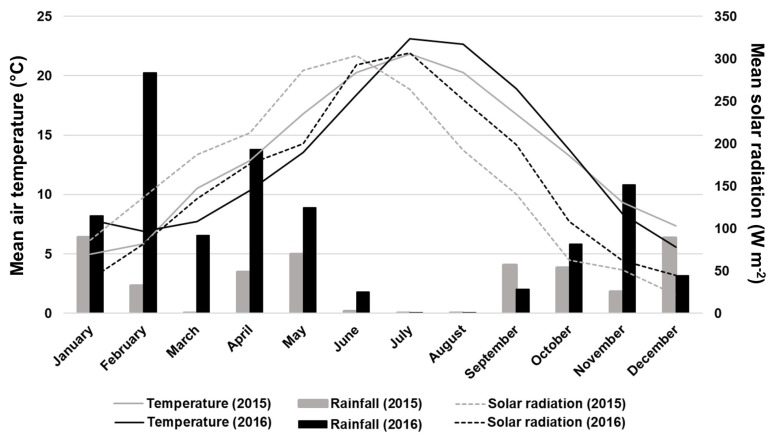
Mean air temperature (°C), rainfall (mm), and mean solar radiation (W m^−2^) in 2015 and 2016 measured at Carrazedo de Montenegro.

Meteorological data [air temperature (°C), rainfall (mm), and solar radiation (W m^−2^)] for both years (Figure 1) were recorded by a standard weather station (IMT280, iMETOS, Weiz, Austria) located near the experimental site. In 2015, the mean air temperature between March and June was, on average, about 2.6 °C higher than 2016. Additionally, the mean solar radiation until June was also higher in 2015 than 2016. Annual rainfall in 2015 was 470 mm against the long-term rainfall (30 years) of 923 mm. The year 2016 experienced higher annual rainfall (1140 mm), mainly in spring (Figure 1), corresponding to the final phase of the flower development and the fruit development of cherry trees.

### 2.2. Experimental Design and Treatments

Six trees from each treatment were selected, a total of 42 trees. In 2015, the experiment included the following treatments: 0.5 mL L^−1^ biostimulant *Ascophyllum nodosum* (AN), 10 mg L^−1^ gibberellic acid (GA_3_), 10 µM abscisic acid (ABA), 1 mM salicylic acid (SA), 1 mL L^−1^ glycine betaine (GB), 5 g kg^−1^ calcium (CaCl_2_), and control (distilled water). In 2016, the same seven treatments were applied to the same trees selected in the previous year, but now including 5 g kg^−1^ CaCl_2_, except for the control treatment. All cherry trees were sprayed with 2.5 L of spraying solution per tree. Wetting agent (1 mL L^−1^) was mixed in control and treatment solutions. Foliar treatments, except CaCl_2_, were applied 30, 49, and 56 days after full bloom (DAFB), corresponding to the shuck split (beginning of fruit development), the transition from green to yellow color, and from yellow to orange color. CaCl_2_ was added at 56, 62, and 69 DAFB, corresponding to the first application at the transition from yellow to orange color and the other applications were applied one week later. 

The gas exchange and relative water content (RWC) determinations were performed at midday on 08 July 2015 and 15 July 2016 (corresponding to the harvest date of the cherries) in healthy, fully expanded mature leaves that were well exposed to the sun. For each treatment, fifty fruits were harvested per tree to determine cracking index, and yield was determined for each tree. Furthermore, leaves were collected for biochemical analyses (photosynthetic pigments, total soluble sugars, starch, and soluble proteins) and immediately frozen in liquid nitrogen and then stored at −80 °C until analysis. 

### 2.3. Leaf Gas Exchange 

Leaf gas exchange measurements were performed using a portable LCpro+ Infrared Gas Analyzer System (IRGA) (ADC Bioscientific Ltd, Hoddesdon, England), with a 2.5 cm^2^ leaf chamber (ADC-PLC), operating in the open mode, at midday (13:00–14:30 h) in both years. Incident photosynthetic photon flux density (PPFD) on the leaves was always greater than 1500 μmol m^−2^ s^−1^. Net CO_2_ assimilation rate (*A,* μmol m^−2^ s^−1^), transpiration rate (*E*, mmol m^−2^ s^−1^), and stomatal conductance (*g_s_*, mmol m^−2^ s^−1^) were calculated using the equations developed by von Caemmerer and Farquhar [33]. Intrinsic water-use efficiency was calculated as the ratio of *A* to *g_s_* (*A/g_s_*, µmol mol^−1^), according to Düring [34]. All results are expressed as the average of six replicates with standard error (SE) shown.

### 2.4. Leaf Water Status 

After the midday gas exchange measurements, sweet cherry leaves were detached and immediately placed into air-tight tubes, and the following parameters were studied: fresh weight (FW in g), weight at full turgor (TW in g, measured after immersing the leaf petioles in deionized water for 24 h at 4 °C in the dark), and dry weight (DW in g, measured after drying at 70 °C to a constant weight). The relative water content (RWC in %) was calculated as follows: RWC = (FW−DW)/(TW−DW) × 100. Results are expressed as the average of six replicates with SE shown.

### 2.5. Metabolite Composition Determination 

#### 2.5.1. Photosynthetic Pigments 

For chlorophyll (Chl) and carotenoid (Carot) determination, leaf discs (0.8 cm diameter) were ground with mortar and pestle using acetone/distilled-water (80/20, *v/v*) as extraction solvent. Analyses were performed under the dim light to avoid chlorophyll degradation. Determination of total chlorophyll (Chl_total_) and total carotenoids (Carot_total_) were performed according to Šesták et al. [35] and Lichtenthaler [36], respectively. The results were expressed as mg g^−1^ DW as the mean ± SE of six replicates.

#### 2.5.2. Total Soluble Sugars and Starch

Total soluble sugars (SS) quantification was performed using the methodology of Irigoyen et al. [37], by heating foliar discs in ethanol/distilled-water (80/20, v/v) for 1 h, at 80 °C. After the reaction of the alcoholic extract with fresh anthrone in a boiling water bath for 10 min, SS were quantified by recorded absorbance values at 625 nm. Afterwards, starch (St) was extracted from the same solid fraction by heating leaf discs in 30% perchloric acid at 60 °C for 1 h, using the methodology of Osaki et al. [38]. The St concentration was determined by the anthrone method described above. Glucose was used as a standard for both SS and St quantification. The results were expressed as mg g^−1^ DW as the mean ± SE of six replicates.

#### 2.5.3. Soluble Proteins

Total soluble proteins (SP) determination was performed using phosphate buffer (pH 7.5), 0.1 mM ethylenediaminetetraacetic acid (EDTA), 100 mM phenyl-methylsulfonyl fluoride (PMSF), and 20 g L^−1^ polyvinylpyrolli-done (PVP). The absorbance values were recorded at 595 nm, using bovine serum albumin (BSA) as standard [39]. The results were expressed as mg g^−1^ DW as the mean ± SE of six replicates.

### 2.6. Yield Determination

Sweet cherries were harvested at optimum maturity stage and yield per tree was recorded in kilograms as the mean ± SE of six replicates.

### 2.7. Fruit Cracking Index Determination

The cracking index (CI in %) was determined according to Christensen [40]. Fifty fruits without defects were selected and immersed in 2 L plastic containers filled with distilled water (20 ± 1 °C) for 6 h. Cracked fruits were removed, counted, and fruits without cracks were reincubated. After 2, 4, and 6 h, the fruits were observed for macroscopic cracks, with the CI calculated according to the following formula: CI = [(5a + 3b + c) × 100]/250, where a, b, and c represent the number of cracked fruits after 2, 4, and 6 h, respectively. The measurements are presented as average values (*n* = 3) with SE.

### 2.8. Statistical Analysis

The statistical analysis was carried out using the statistical software program SPSS V.25 (SPSS-IBM, Orchard Road-Armonk, New York, NY, USA). Statistical differences were evaluated by one-way analysis of variance (ANOVA) followed by the post hoc Duncan’s multiple range test (*P* < 0.05), establishing treatment effect. The ANOVA requirements, namely the normal distribution of the residuals and the homogeneity of variance, were evaluated by means of the Shapiro–Wilk’s test and Bartlett’s tests, respectively. Dependent variables were analyzed using ANOVA with or without Welch correction, depending if homogeneity of variances was observed or not. For the relationship between parameters, Pearson’s correlation was performed. 

## 3. Results

### 3.1. Leaf Gas Exchange Parameters

**Figure 2:**
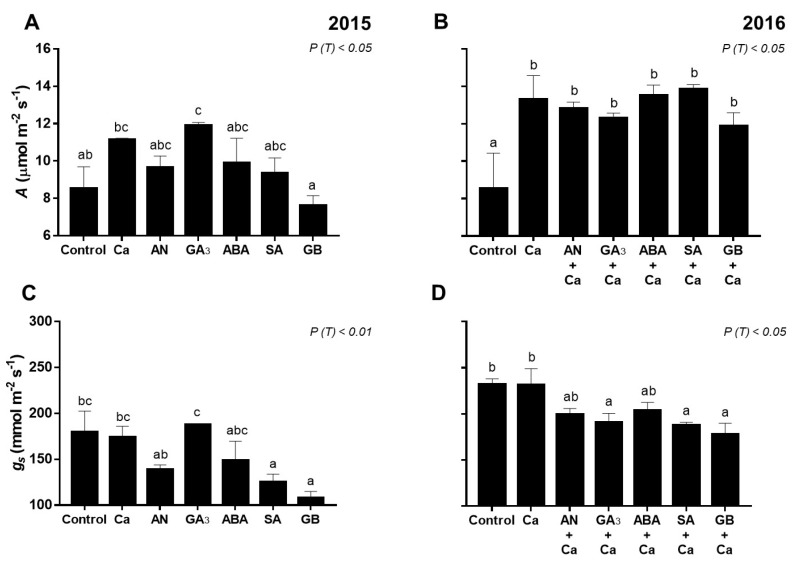

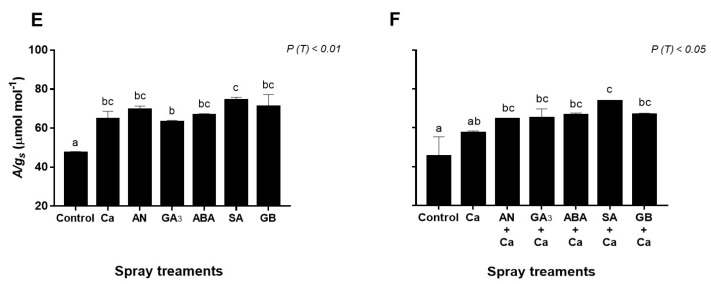
Net CO_2_ assimilation (*A*) (**A**,**B**), stomatal conductance (*gs*) (**C**,**D**), and intrinsic water-use efficiency (*A/gs*) (**E**,**F**) of fully exposed leaves of ‘Skeena’ cherry treated after spray treatments application in 2015–2016. Each column is expressed as mean ± SE (n = 6). Different letters indicate significant differences (*P* < 0.05) among treatments by Duncan’s test.

The results show that for both years, most of the gas exchange parameters were significantly affected (*P* < 0.05) by the spray treatments (Figure 2), except the transpiration rate (E) (data not shown). 

In 2015, GA_3_ sprays increased (*P* < 0.05) photosynthetic rate (*A*) compared to the control (H_2_O) (Figure 2a). In turn, SA and GB spray treatments decreased (*P* < 0.01) *g_s_* (Figure 2c). All spray treatments increased (*P* < 0.01) intrinsic water-use efficiency (*A/g_s_*) compared to the control (Figure 2e). 

In 2016, spray treatments increased (*P* < 0.05) *A*, with the highest value for SA+Ca-treated cherry trees (13.85 µmol m^−2^ s^−1^), whereas control trees recorded the minimum rate (Figure 2b). GA_3_+Ca-, SA+Ca-, and GB+Ca-treated cherry trees presented significantly lower (*P* < 0.05) *g_s_* values than control and Ca-treated cherry trees (Figure 2d). AN+Ca, GA_3_+Ca, ABA+Ca, SA+Ca, and GB+Ca spray treatments increased (*P* < 0.05) *A/g_s_* compared to the control (Figure 2f).

### 3.2. Leaf Water Status

**Figure 3:**
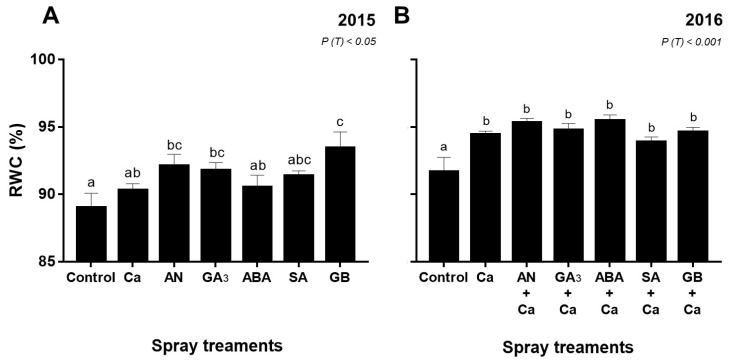
Relative water content (RWC, %) of ‘Skeena’ leaf cherry after spray treatments application in 2015 (**A**) and 2016 (**B**). Each column is expressed as mean ± SE (n = 6). Different letters indicate significant differences (*P* < 0.05) among treatments by Duncan’s test.

In both years, relative water content (RWC) was affected by the spray treatments (*P* < 0.05) (Figure 3). In 2015, treatments with GA_3_, AN, and GB increased (*P* < 0.05) RWC up to 3%, 3.4%, and 4.8%, respectively, compared with the control (Figure 3A). In 2016, all spray treatments presented higher (*P* < 0.001) RWC values in comparison with control plants. The highest value was obtained in cherry trees treated with ABA+Ca, about 4% higher than control (Figure 3B).

### 3.3. Leaf Photosynthetic Pigments and Metabolites 

The content of photosynthetic pigments Chl_total_ and Carot_total_ was affected by spray treatments (*P* < 0.05) in 2015, while in 2016 significant differences (*P* < 0.05) were only found for the content of Chl_total_ (Table 1). Compared with the control, the highest Chl_total_ content (10.56 mg g^−1^ DW) was observed with the ABA treatment in 2015 and both AN+Ca and GB+Ca treatments in 2016. In relation to the Carot_total_ content in 2015, cherry trees treated with Ca, AN, GA_3_, SA, and GB presented higher (*P* < 0.001) values than the control. Both years presented similar Chl_total_ contents for control and Ca-treated plants. In turn, Carot_total_ concentration seems to be influenced by the year, being higher (*P* < 0.01) in 2016. 

The results also indicated that spray treatments increased leaf metabolites in cherry leaves (Table 1). Soluble sugars (SS) content was significantly affected by the spray treatments (*P* < 0.05) in both years. In 2015, AN and ABA sprays increased (*P* < 0.05) SS content in relation to the control. On the other hand, addition of Ca to GA_3_, ABA, and SA usually resulted in higher SS concentration in 2016. Significant differences were also observed among spray treatments for soluble proteins (SP) and starch (St) concentration in the first and second year of the experiment, respectively. In 2015, the highest SP concentration was recorded in cherry trees sprayed with AN, GA_3_, SA, and GB. In 2016, the combined treatments AN+Ca, ABA+Ca, SA+Ca, and GB+Ca showed a higher (*P* < 0.01) St content.

### 3.4. Yield of Sweet Cherry Trees

**Figure 4:**
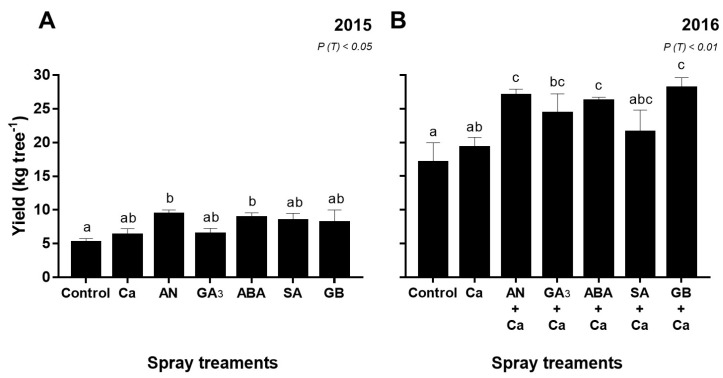
Yield (kg tree^−1^) of ‘Skeena’ cherry trees after spray treatments application in 2015 (**A**) and 2016 (**B**). Each column is expressed as mean ± SE (n = 6). Different letters indicate significant differences (*P* < 0.05) among treatments by Duncan’s test.

Significant yield differences were found among spray treatments (*P* < 0.05) in both years (Figure 4). In 2015, cherry trees sprayed with AN and ABA exhibited the highest (*P* < 0.05) yield, averaging 45% and 41%, respectively, compared to the control (Figure 4A). This behavior was also observed in 2016, although the highest production was obtained for GB+Ca-treated cherry trees, up to 40% compared to the control (Figure 4B). 

### 3.5. Cracking Incidence

**Figure 5:**
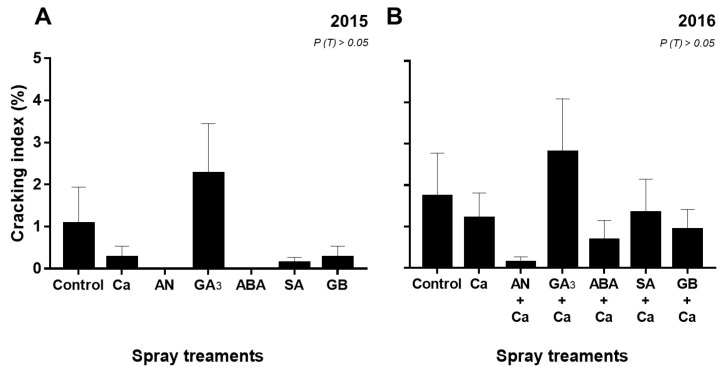
Cracking index (%) of ‘Skeena’ fruits after spray treatments application in 2015 (**A**) and 2016 (**B**). Each column is expressed as mean ± SE (n = 3, each with 50 fruits).

Spray treatments did not affect significantly (*P* > 0.05) the fruit cracking index (CI) in both years (Figure 5). However, cherries treated with Ca, AN, ABA, SA, and GB spray treatments showed a decreasing trend of CI in both years, compared to the control. In contrast, GA_3_/GA_3_+Ca sprays increased the CI, in both years.

## 4. Discussion

### 4.1. Spray Treatments Modulate Leaf Gas Exchange and Water Status of Sweet Cherry Trees

Overall, spray treatments improved the physiological behavior of sweet cherry trees in both years (Figure 2). *A* presented a positive correlation with *g_s_* (*r* = 0.54, *P* < 0.001), as observed previously by Gonçalves et al. [41] in sweet cherry. Higher *g_s_* values were noticed in 2016 compared to 2015, which may be due to the higher solar radiation recorded in July 2016, near the harvest, and consequently, the values of *A* were also higher in 2016 (Figure 1 and Figure 2). Our findings are in agreement with previous studies, which demonstrated an increase of *A* in broad beans (*Vicia faba*) and grapevine (*Vitis vinifera* L.) treated with GA_3_ [42]. Grapevine treated with GA_3_ also resulted in favorable *A/g_s_* [43]. Other studies with *Brassica juncea*, corn, and soybean treated with SA also showed an improvement of *A*, *A/g_s_* and *g_s_* adjustment [44,45,46], which agrees with our results. The first sign of plant defense for maintaining the water status is the stomatal closure [47]. Indeed, our results indicate that GB sprays improved *A* and also increased stomata closure by the reduction of *g_s_* (Figure 2), which might be considered a strategy for enhancing tolerance to various abiotic stresses [48]. Moreover, GB-treated olive trees under drought showed an enhancement of *A* [49]. *Tradescantia virginiana* plants treated with ABA and grown under well-watered conditions had lower *g_s_* and an improvement in *A/g_s_*, while the *A* was unaffected [50]. Although, in both years of our study, the application of ABA increased *A/g_s_*, *g_s_* was not affected, and an improvement in *A* was observed, mainly in 2016. Additionally, *A* and *A/g_s_* values also increased in response to AN+Ca treatment (in 2016) (Figure 2). This finding is in agreement with previous works, which revealed an increase of *A*, a reduction of *E*, and *g_s_* parameters in plants treated with biostimulant products [29,30].

The leaf RWC was around 90% in both years, suggesting sufficient drip irrigation. Nevertheless, in treated-plants with foliar compounds, the RWC values were mostly higher in both years (Figure 3). The water balance in plants is estimated by calculation of RWC, which is positively correlated with the photosynthetic efficiency of plants [51]. This relationship is established by the correlation between RWC and *A*, mainly in 2016 (*r* = 0.54, *P* < 0.05). Similarly with our results, a significant improvement of RWC in different crops exposed to several biotic stresses and treated with biostimulant [29,52,53,54], GA_3_ [55], ABA [56], SA [57], and GB [49,58] was previously reported.

### 4.2. Photosynthetic Pigments’ and Metabolites’ Behavior in Response to Spray Treatments

Chl_total_ exhibited the same tendency as *A*, displaying higher contents compared to the control in both years (Table 1). ABA sprays increased Chl_total_ content, which is in accordance with a previous study that revealed that the exogenous application of ABA increases the synthesis of chlorophylls in ‘micro’ tomato leaf tissue [59]. Photosynthetic pigments contents, Chl_total_ and Carot_total_, also increased in AN-, SA-, and GB-treated cherry trees (Table 1). Similarly, Kabiri et al. [60], mentioned that the SA application in *Nigella sativa* increased the content of chlorophylls and carotenoids. According to Hayat and Ahmad [61], this increase improved the antioxidant capacity of plants and it was related to the synthesis of protective compounds. Additionally, overaccumulation of GB due to the introduction of the *betaine aldehyde dehydrogenase* (BADH) gene can increase the protection of chlorophylls and carotenoids and enhance the photosynthetic rate [62]. Several biostimulants, like AN, have been stated to stimulate plant growth by increasing photosynthetic pigments [28]. These findings can be related to the preservation of carotenoids as a mechanism of photoprotection [63] and amelioration of the leaf water retention. Our study indicates that spray treatments, mainly AN, SA, and GB induced the increased chlorophylls and carotenoids levels in the leaf tissue, which can improve the antioxidant capacity of plants to abiotic stress. 

The spray treatments also affected the SP content in cherry leaves, showing higher levels in AN-, GA_3_-, SA-, and GB-treated trees, up to 34% with SA spray treatment (Table 1). These treatments might induce the growing of antioxidant responses as described for SA [18,61]. The higher accumulation of SS in leaves observed in response to AN and ABA sprays in 2015 and to GA_3_+Ca, ABA+Ca, and SA+Ca sprays in 2016 (Table 1) can be a protective mechanism to preserve cell homeostasis, indicating that these spray treatments provided a better cherry tree photosynthetic performance. Interestingly, the highest SS accumulation in cherry leaves was found for ABA spray treatment for around 20% in both years (Table 1). Exogenous application of ABA is reported to increase the maturity index and anthocyanin content in cherries [64] and the soluble sugars in grapes [65]. Although our previous works reported that ABA application increased anthocyanin content in cherry, no significant effect was observed on the maturation [66]. The highest St content observed in 2016 might be related to the higher photosynthetic efficiency determined in the same year (Table 1). Overall, the present study suggests a relation between acclimation of photosynthesis (*A*) and St accumulation (*r* = 0.46, *P* < 0.05). Indeed, several authors have suggested that a decrease of St in leaf was correlated with the acclimation of photosynthesis [67,68]. In 2016, the higher accumulation of St in mainly AN+Ca-, ABA+Ca-, SA+Ca-, and GB+Ca-treated cherry trees may have a positive effect on tree production, since St is a crucial storage carbohydrate that is frequently mobilized in the form of SS [69]. The St accumulation in leaves of ABA+Ca-, SA+Ca-, and GB+Ca-treated cherry trees could be associated with the increase in weight observed in the fruits collected from the plants treated with these spray treatments [23]. In addition, ABA might be related to control of the enzymes responsible for St degradation, regulating the St accumulation during osmotic stress in plants [70].

### 4.3. Effect of Spray Treatments on Yield of Sweet Cherry Trees and Fruit Cracking Incidence

Yield and cracking incidence evaluation are important parameters in the effectiveness of spray treatments. However, fruit yield is a function of several factors, such as meteorological conditions, rootstock vigor, irrigation, and pruning [71]. Our previous work reported that on average, fruit weight was reduced in 2016 by 33% compared to 2015 [23]. On the other hand, yield was higher in 2016 compared to 2015 (Figure 4), which was due to a higher crop load rather than larger fruit size. This may also be because of a winter pruning in 2014, which will have reduced the number of flowers in the following year (2015); in turn, in 2015 no pruning was performed. Nevertheless, AN and ABA sprays were related to the increase in yield in both years, while in 2016 also GA_3_- and GB-treated cherry trees showed a significant increase of yield (Figure 4). Although cherry cracking incidence did not significantly decrease with spray treatments in both years, it was observed that also the AN- and ABA-treated fruits showed the least cracking index in 2015 and 2016 (Figure 5). Reduced cracking index for ABA-treated ‘Bing’ cherry was reported by Balbontín et al. [25]. An additional promising strategy to mitigate cherry cracking is AN, as had been reported by our research group for ‘Skeena’ and ‘Sweetheart’ cherry fruits [31]. On the other hand, foliar application of GA_3_ increased ‘Skeena’ cherry cracking, and this finding corroborates with previous studies [12]. Biostimulants, like AN, have offered a potentially novel approach in plants to stimulate growth and to increase yield, as reported by Basak [72], Colavita et al. [73], and Jannin et al. [30]. Quiroga et al. [74] reported that ABA foliar application in the field-grown grapevine could improve yield per plant. Our data suggest that the application of GA_3_ and GB seems to benefit with the combination of Ca in increasing of cherry yield. A positive impact of exogenous GB application on plant growth and final crop yield has been reported on sunflower (*Helianthus annuus* L.) and maize (*Zea mays*) under drought [75,76]. As observed in our study, foliar application of GA_3_ increased fruit yield in several crops, such as in tomato [77,78] and cucumber (*Cucumis sativus* L.) [79]. Our data also indicated a positive correlation between yield and *A* (*r* = 0.54, *P* < 0.01), as observed by Parry et al. [80] and Zhu et al. [81] in other species. Positive correlations were also observed between yield and SS (*r* = 0.50, *P* < 0.001), St (*r* = 0.83, *P* < 0.001), SP (*r* = 0.46, *P* < 0.01), and RWC (*r* = 0.66, *P* < 0.001). Indeed, other studies have also reported that yield is correlated positively with SS and RWC in *Sorghum bicolor* L. [82] and in several cultivars of banana fruits [83]. 

## 5. Conclusions

Foliar spraying of growth regulators and calcium was associated with an enhancement of the physiological performance and yield of the ‘Skeena’ sweet cherry trees. Among the treatments evaluated, foliar application of AN and ABA were more effective in increasing yield and in reducing the incidence of cherry cracking. Therefore, these two foliar sprays are attractive compounds to improve physiological performance and yield of sweet cherry trees and might be a promising cherry-cracking mitigation approach. Although AN and ABA foliar application might be a cultural practice in the near future, further studies on the influence of environmental conditions, concentrations, and time of foliar compounds application in order to develop new cultivar-specific farming strategies will be required.

## Figures and Tables

**Table 1:** Total chlorophyll content (Chl_total_), total carotenoids content (Carot_total_), soluble sugars content (SS), starch content (St), and soluble protein content (SP) of ‘Skeena’ leaf cherry after spray treatments application in 2015–2016.

Year	Treatment (T)	Chl_total_ (mg g^−1^ DW)	Carot_total_ (mg g^−1^ DW)	SS (mg g^−1^ DW)	St (mg g^−1^ DW)	SP (mg g^−1^ DW)
**2015**	**Control**	7.95 ± 0.42 a	0.93 ± 0.04 a	70.35 ± 1.16 a	42.98 ± 0.56 a	10.34 ± 0.79 a
	**Ca**	8.37 ± 0.86 a	1.15 ± 0.05 cd	75.04 ± 3.87 abc	41.67 ± 1.94 a	12.30 ± 0.53 ab
	**AN**	9.34 ± 0.27 ab	1.23 ± 0.05 de	85.81 ± 3.00 bc	47.41 ± 1.89 a	15.08 ± 0.43 cd
	**GA_3_**	9.10 ± 0.32 ab	1.35 ± 0.04 e	75.24 ± 4.85 abc	45.39 ± 2.59 a	13.81 ± 0.91 bcd
	**ABA**	10.56 ± 0.68 b	1.01 ± 0.02 ab	87.44 ± 4.65 c	48.13 ± 3.00 a	12.90 ± 1.08 abc
	**SA**	8.56 ± 0.33 a	1.19 ± 0.05 cd	78.31 ± 6.03 abc	49.94 ± 1.39 a	15.75 ± 1.06 d
	**GB**	8.35 ± 0.26 a	1.10 ± 0.03 bc	72.79 ± 4.63 ab	48.06 ± 2.91 a	15.26 ± 1.11 cd
	***P (T)***	*	***	*	*ns*	**
**2016**	Control	7.51 ± 0.26 ab	1.19 ± 0.14 a	75.85 ± 2.12 a	101.10 ± 2.10 a	10.79 ± 0.69 a
	**Ca**	7.24 ± 0.40 a	1.25 ± 0.05 a	80.84 ± 3.74 ab	116.49 ± 6.77 ab	10.87 ± 0.85 a
	**AN+Ca**	8.76 ± 0.44 c	1.35 ± 0.04 a	85.89 ± 5.36 ab	126.44 ± 3.28 bc	11.90 ± 0.79 a
	**GA_3_+Ca**	7.97 ± 0.78 abc	1.30 ± 0.07 a	91.60 ± 1.85 b	110.58 ± 5.14 ab	12.90 ± 1.41 a
	**ABA+Ca**	8.22 ± 0.40 abc	1.32 ± 0.05 a	93.92 ± 5.09 b	121.41 ± 8.12 bc	13.29 ± 0.54 a
	**SA+Ca**	8.50 ± 0.43 bc	1.34 ± 0.05 a	93.27 ± 6.69 b	137.61 ± 7.71 c	11.64 ± 0.92 a
	**GB+Ca**	8.86 ± 0.39 c	1.43 ± 0.06 a	87.67 ± 2.71 ab	125.77 ± 7.27 bc	12.32 ± 1.42 a
	***P (T)***	*	*ns*	*	**	*ns*

Values are means ± standard error (n = 6). Means flanked by the same letter are not significantly different at *P* < 0.05 (Duncan’s test). * *P* < 0.05, ** *P* < 0.01, *** *P* < 0.001 by Duncan’s test, *ns*—not significant. DW: Dry Weight.
